# Supplementary material for: In vitro reconstitution reveals major differences between human and bacterial cytochrome c synthases
Source: eLife. 2021 May 11;10:e64891. doi: 10.7554/eLife.64891 (PMC8112865; doi:10.7554/eLife.64891)
Supplement: Supplementary file 1. [file elife-64891-supp1.docx]

**Supplementary File 1: Table S1. Relevant Strains, Plasmids and Primers Employed in this Study**

| *Table S1****.*** *Relevant Strains, Plasmids and Primers Employed in this Study* | | |  | | | |  | | |
| --- | --- | --- | --- | --- | --- | --- | --- | --- | --- |
| Strain, Plasmid | Description | Reference | | | |  | | |  |
| *E. coli* |  |  | | | |  | | |  |
| NEB 5-α | fhuA2 Δ(argF-lacZ)U169 phoA glnV44 Φ80 Δ(lacZ)M15 gyrA96 recA1 relA1 endA1 thi-1 hsdR17 |  | | | |  | | |  |
| C43(DE3) | F – ompT hsdSB (rB- mB-) gal dcm (DE3) | (Miroux and Walker, 1996) | | | |  | | |  |
| RK103 | *E. coli* MG1655 Δ*ccm::kan^R^*, deleted for all *ccm* genes | (Feissner et al., 2006) | | | |  | | |  |
| MS36 | C43 Δ*ccm::kan^R^*, deleted for all *ccm* genes | (Sutherland et al., 2018) | | | |  | | |  |
|  |  |  | | | |  | | |  |
| *Plasmid* |  |  | | | |  | | |  |
| pRGK332 | pBAD *Bordetella pertussis* cytochrome c4:His | (Feissner et al., 2006) | | | |  | | |  |
| pRGK368 | pGEX *Helicobacter hepaticus* GST:CcsBA | (Richard-Fogal et al., 2007) | | | |  | | |  |
| pRGK403 | pGEX GST:HCCS | (San Francisco et al., 2013) | | | |  | | |  |
| pRGK420 | pGEX GST:HCCS H154A | (San Francisco et al., 2013) | | | |  | | |  |
| pMCS97 | pGEX *H. hepaticus* GST:CcsBA:His | This study | | | |  | | |  |
| pMCS64 | pGEX *H. hepaticus* GST*CcsBA:His | This study | | | |  | | |  |
| pMCS154 | pGEX *H. hepaticus* GST:CcsBA P-His1/2G | (Sutherland et al., 2018) | | | |  | | |  |
| pMCS558 | pGEX *H. hepaticus* *CcsBA:His | This study | | | |  | | |  |
| pMCS598 | pGEX *H. hepaticus* GST*CcsBA:His P-His1/2G | This study | | | |  | | |  |
|  |  |  | | |  | | |  |  |
| Oligonucleotide | Sequence (5' --> 3') | Purpose | | Template | | | |  |  |
| pGEX GST*F | tcggatctggttccgcgttgaaggaggaaggatccatgatgaat | pMCS64 cloning | | pMCS97 | | | |  |  |
| pGEX GST*R | attcatcatggatccttcctccttcaacgcggaaccagatccga | pMCS64 cloning | | pMCS97 | | | |  |  |
| pGEX CcsBA 6HisF | gagtgcttgatatgccccatttacatcaccatcaccatcactaactcgagcggc | pMCS97 cloning | | pRGK368 | | | |  |  |
| pGEX CcsBA 6HisR | gccgctcgagttagtgatggtgatggtgatgtaaatggggcatatcaagcactc | pMCS97 cloning | | pRGK368 | | | |  |  |
| MSP5 | gtgcttaaatcttattggctcaacattggcgtctccgtcatca | pMCS598 cloning | | pMCS64 | | | |  |  |
| MSP6 | tgatgacggagacgccaatgttgagccaataagatttaagcac | pMCS598 cloning | | pMCS64 | | | |  |  |
| MSP7 | ttattatctcacaggtatgggcagctatgccgcaggagaa | pMCS598 cloning | | pMCS64 | | | |  |  |
| MSP8 | ttctcctgcggcatagctgcccatacctgtgagataataa | pMCS598 cloning | | pMCS64 | | | |  |  |
