# Supplementary material for: In vitro reconstitution reveals major differences between human and bacterial cytochrome c synthases
Source: eLife. 2021 May 11;10:e64891. doi: 10.7554/eLife.64891 (PMC8112865; doi:10.7554/eLife.64891)
Supplement: Supplementary file 2. [file elife-64891-supp2.docx]

**Supplementary File 2: Table S2. Apo peptides used for *in vitro* assays**

| *Table S2****.*** *Apo peptides used in the in vitro assay* | | |
| --- | --- | --- |
| Peptides | Sequence | Company, Purity |
| Horse heart cytc | **…GDVEKGKKIFVQKCAQCHTVE…** | Sigma, chemically stripped of heme |
| Biotin-56-mer | **Biotin-Ahx-GDVEKGKKIFIMKCSQCHTVEKGGK HKTGPNLHGLFGRKTGQAPGYSYT AANKNKG** | RS-synthesis, 84.98%(by HPLC), ms data conforms |
| Biotin-20 mer | **Biotin-Ahx-GDVEKGKKIFIMKCSQCHTV** | RS-synthesis, 89.42%(by HPLC), ms data conforms |
| 20-mer | **GDVEKGKKIFIMKCSQCHTV** | RS-synthesis, 98.00%(by HPLC), ms data conforms |
| Biotin-16 mer | **Biotin-KGKKIFIMKCSQCHTV** | RS-synthesis, 93.97%(by HPLC), ms data conforms |
| 11 mer | **IMKCSQCHTVE** | RS-synthesis, 98.09%(by HPLC), ms data conforms |
| 9 mer-biotin | **Biotin-Ahx-KCSQCHTVE** | RS-synthesis, 90.88%(by HPLC), ms data conforms |
| 20 mer Cys15S | **GDVEKGKKIFIMKSSQCHTV** | RS-synthesis, 87.63%(by HPLC), ms data conforms |
| 20 mer DCys15 | **GDVEKGKKIFIMK(D-C)SQCHTV** | CS Bio Co, 95.22%(HPLC), ms data conforms |
| 20 mer HoCys15 | **GDVEKGKKIFIMKHoCSQCHTV** | CS Bio Co, 81.55%(HPLC), ms data conforms |
| 20 mer Cys18S | **GDVEKGKKIFIMKCSQSHTV** | CS Bio Co, 94.74%(HPLC), ms data conforms |
| 20 mer DCys18 | **GDVEKGKKIFIMKCSQ(D-C)HTV** | CS Bio Co, 91.00%(HPLC), ms data conforms |
| 20 mer HoCys18 | **GDVEKGKKIFIMKCSQHoCHTV** | CS Bio Co, 85.04%(HPLC), ms data conforms |
| 20 mer Cys15S/Cys18S | **GDVEKGKKIFIMKSSQSHTV** | CS Bio Co, 86.55%(HPLC), ms data conforms |
| 20mer H19A | **GDVEKGKKIFIMKCSQCATV** | RS-synthesis, 86.41%(by HPLC), ms data conforms |
| 20mer H19M | **GDVEKGKKIFIMKCSQCMTV** | RS-synthesis, 93.80%(by HPLC), ms data conforms |
| 20mer H19K | **GDVEKGKKIFIMKCSQCKTV** | RS-synthesis, 86.13%(by HPLC), ms data conforms |
| 20mer K6A, K8D, K9D, K14D | **GDVEAGDDIFIMDCSQCHTV** | RS-synthesis, 87.13%(by HPLC), ms data conforms |
